# Combined effects of reproductive and hormone factors and obesity on the prevalence of knee osteoarthritis and knee pain among middle-aged or older Chinese women: a cross-sectional study

**DOI:** 10.1186/s12889-018-6114-1

**Published:** 2018-10-22

**Authors:** Min Zhou, Jianghao Chen, Dongming Wang, Chunmei Zhu, Youjie Wang, Weihong Chen

**Affiliations:** 1 Key Laboratory of Environment and Health in Ministry of Education &amp; Ministry of Environmental Protection, and State Key Laboratory of Environmental Health (Incubating), School of Public Health, Tongji Medical College, Huazhong University of Science and Technology, Hangkong Road 13, Wuhan, 430030 Hubei China; 2 Department of Occupational and Environmental Health, School of Public Health, Tongji Medical College, Huazhong University of Science and Technology, Hangkong Road 13, Wuhan, 430030 Hubei China; 3 Department of Maternal and Child Health, School of Public Health, Tongji Medical College, Huazhong University of Science and Technology, Hangkong Road 13, Wuhan, 430030 Hubei China

**Keywords:** Female, Knee pain, Knee osteoarthritis, Reproductive and hormone factors, Obesity

## Abstract

**Background:**

Knee osteoarthritis (KOA) is one form of degenerative arthritis that results from the breakdown of cartilage and underlying bone. The prevalence of KOA is considerably higher in women than in men; however, the reason for this difference has not been thoroughly elucidated to date. The aim of the present study was to estimate the effects of reproductive and hormone factors and obesity on KOA prevalence among Chinese women.

**Methods:**

The cross-sectional study included 7510 women with a mean age of 62.6 ± 8.6 years. Knee pain was defined as pain or aching stiffness on most days for at least 1 month during the past 12 months or persistent pain or aching stiffness within the past week. Clinical KOA was diagnosed based on both pain complaints and a Kellgren-Lawrence grade ≥ 2 X-ray radiograph of at least one knee.

**Results:**

Oral contraceptives use (OR 1.18, 1.05–1.34), ≥3 pregnancies (1.38, 1.20–1.60), and postmenopausal hormone replacement therapy (HT) (1.59, 1.23–2.06) were positively associated with knee pain, while oral contraceptives use (1.28, 1.04–1.57), and HT (1.79, 1.21–2.65) were positively associated with clinical KOA. Obesity and oral contraceptives use showed additive and multiplicative effects on knee pain. The OR for knee pain among women with a BMI ≥24 kg/m^2^ and oral contraceptives use was 2.00 (1.68–2.38) compared with women with a BMI &lt; 24 kg/m^2^ and no oral contraceptives use.

**Conclusions:**

A high number of pregnancies, oral contraceptives use, and HT are independent risk factors for KOA, and the effects of reproductive and hormone factors on KOA may be increased by obesity.

## Background

Knee osteoarthritis (KOA) is one form of degenerative arthritis that results from the breakdown of cartilage and underlying bone. A high prevalence of clinical KOA, ranging from 5.6 to 28.9%, has been reported across regions and countries [1–3]. Knee pain is the most common presenting feature of KOA. Disability associated with knee pain affects up to 10% of adults aged 55 or older [4]. Lower limb disability following KOA is thought to be an important cause of poor quality of life and work limitations. KOA is reported to be one of the largest economic burdens worldwide [5, 6].

Accumulating evidence suggests that the prevalence of KOA is much higher in women than in men. The results from the Johnston County (North Carolina, USA) Osteoarthritis Project reported that the prevalence of symptomatic KOA among women and men was 17.6% and 14.5%, respectively [3]. The China Health and Retirement Longitudinal Study showed that the prevalence of symptomatic KOA was higher in women (10.3%) than that in men (5.7%) [7]. The reason for the higher prevalence of KOA in women remains unclear. In addition to physical characteristics, female-specific physiological phenomenon may play an important role in the development and progression of KOA. Currently, several papers have indicated that reproductive and hormone factors may contribute to the elevated prevalence of KOA among females. Szoeke and colleagues found that hormone replacement therapy was a risk factor for KOA in a study of 224 Australian women [8]. A multicenter osteoarthritis study with 1618 women reported that an increased number of pregnancies was positively associated with an elevated incidence of KOA [9]. Alternations in sex hormones might affect bone turnover via the estrogen receptors in the musculoskeletal system and disturb the bone and cartilage metabolic balance [10, 11]. Reproductive and hormone factors are also associated with obesity [12, 13], with the latter being reported to increase knee joint burden [14]. However, whether obesity modifies the association between reproductive and hormone factors and KOA has not been adequately examined to date.

The objective of this study was to evaluate the independent and combined effects of reproductive and hormone factors and obesity on the prevalence of knee pain and clinical KOA among middle-aged and older Chinese females.

## Methods

### Design

The study design was cross-sectional.

### Participants

The participants in the present study came from Dongfeng-Tongji cohort that was reported previously [15]. Questionnaires were administered face-to-face by trained interviewers to collect information on demographics, medical history, lifestyle, occupational history, and reproductive and hormone factors. After the interviews, all participants underwent physical examinations. A total of 14,438 participants, i.e., those that underwent physical examinations at the Central Hospital of the Dongfeng Motor Corporation, had their knees examined (for tenderness, range of motion, extension test, and McMurray’s test) and were questioned about knee symptoms (pain or aching stiffness in or around the knee). After excluding individuals with missing data regarding knee health status (*n* = 182) or women with missing information regarding reproductive and hormone factors (*n* = 50), the response rate was 98.4%. Participants with knee surgery caused by accidental injury, which was associated with secondary KOA (*n* = 247), or with a history of rheumatoid arthritis (*n* = 103), or male gender (*n* = 6346) were further excluded, and 7510 women were included in the final analysis.

### Assessment of knee pain and clinical KOA

Knee pain was diagnosed when any knee met the following criteria: 1) pain or aching stiffness in or around the knee on most days for at least 1 month during the past 12 months and/or 2) persistent pain or aching stiffness in or around the knee within the past week.

Information regarding self-reported clinically diagnosed KOA was collected from the questionnaires and confirmed by insurance records and treatment information. Clinical KOA was diagnosed only if the patient had knee pain complaints and bilateral weight-bearing anteroposterior X-ray radiographs showing a Kellgren and Lawrence grade ≥ 2 for at least one knee [16].

### Assessment of reproductive and hormone factors

Information regarding reproductive and hormone factors was collected from the questionnaires. Pregnancy times were defined as the total number of pregnancies and were categorized into three subgroups: ≤1, 2, and ≥ 3, and the limited number of nulliparous women were categorized as a ≤ 1 group (*n* = 60). Abortion was defined as terminating pregnancy before the 28th gestational week or before the fetus weighed 1000 g. Menopause was defined as no vaginal bleeding for at least one year. The duration of post-menopausal status was categorized as 0 (pre-menopause), &lt; 9, 9–17, and ≥ 17 years. Information on past oral contraceptives use and hormone replacement therapy (HT) history was collected by asking “Have you ever taken any oral contraceptive drugs?” and “Have you ever used any hormone replacement therapy?”

### Assessment of obesity

Body height and weight were measured to the nearest 0.1 cm and 0.1 kg with participants wearing light indoor clothing. Body mass index (BMI) was calculated from weight in kilograms divided by the body height in square meters and was categorized into normal bodyweight or below (&lt; 24.0 kg/m^2^), overweight (24.0–28.0 kg/m^2^), or obese (≥28.0 kg/m^2^) according to World Health Organization recommended BMI cut-off points for Asian populations [17].

### Assessment of covariates

Information on age, gender (male/ female), education level (elementary or lower/ junior high school/ high school/ college or higher), drinking status (current/ former/ never), physical exercise (yes/ no), and occupational history (job titles, calendar years of each job, job content) was collected from questionnaires.

Participants who had been drinking alcohol as often as once per week for at least 6 months were considered to be current drinkers. Physical activity was defined as regular leisure time exercise of at least 20 min per day over the past 6 months.

Shift work was identified as working with a schedule involving unusual working hours as opposed to the normal daytime work schedule, i.e., from 8:00 to 17:00 in China, for at least one year. There were 2 common types of shift work in Dongfeng Motor Corporation: (1) two-shifts, day work (8:00–20:00) and night work (20:00-next 8:00) shifted in weekly rotation; (2) three-shifts, during which 3 crews of workers succeed each other at 8:00, 16:00, and 00:00. The workers in any kind of shift work were required to take turns working in the early morning and at night.

Based on the job held for the longest duration, work posture was grouped into sitting, standing, squatting or kneeling, or bending if the participant accumulatively held the posture for half the time or longer during each shift (i.e., ≥4 h for most cases) according to the description of the job content.

### Statistical analysis

Basic characteristics of participants were presented as the means (standard deviation, SD) compared using Student′s t-test for continuous variables and as numbers (percentages) compared using the Chi-square test for categorical variables. Logistic regression models were used to calculate adjusted odds ratios (ORs) and 95% confidence intervals (CIs) for related factors associated with knee pain or clinical KOA. Model 1 adjusted for age and BMI, because previous research suggested that they were important risk factors for KOA [18]. Model 2 further adjusted for education, physical exercise, alcohol drinking, work posture, and shift work, in the event they might affect knee health status or hormone levels [19, 20]. To explore the influence of multiple independent factors on knee pain or clinical KOA, we used multivariable logistic regression with a stepwise selection procedure, starting with variables of interest (including times of pregnancy, abortion, post-menopause duration, HT, contraceptives use, age, BMI, education, physical exercise, alcohol drinking, work posture, and shift work) and ending with variables that were statistically significantly associated with a *p*-value &lt; 0.05. The multiplicative effects of obesity and reproductive and hormone factors on knee pain and clinical KOA were calculated using multivariable logistic regression analyses in which the multiplicative interaction terms were added. To evaluate the additive interaction, the attributable proportion of interaction (AP) were calculated in an additive interaction model [21]. All statistical analyses were performed using SAS 9.4 software (SAS Institute, Cary, NC, USA).

## Results

The basic characteristics of the participants were shown in Table 1. The mean age of participants was 62.6 ± 8.6 years old. The prevalence of knee pain and clinical KOA was 46.6% (*n* = 3496) and 8.5% (*n* = 640), respectively. Participants with knee pain or clinical KOA were of older age, higher BMI, had more pregnancies, a longer post-menopause duration, and more use of HT than their unaffected colleagues. No differences in alcohol drinking or workload were observed between participants with or without knee pain or clinical KOA.

**Table 1:** Basic characteristics of the participants (*n* = 7510)

Variables	Knee pain			Clinical KOA		
	Yes (*n* = 3496)	No (*n* = 4014)	*p-*value	Yes (*n* = 640)	No (*n* = 6870)	*p-*value
Age (years, mean ± SD)	63.1 ± 8.5	61.1 ± 8.6	&lt; 0.01*	65.1 ± 8.6	61.9 ± 8.5	&lt; 0.01*
BMI (kg/m^2^, mean ± SD)	24.7 ± 3.6	23.8 ± 3.3	&lt; 0.01*	25.3 ± 3.8	24.1 ± 3.4	&lt; 0.01*
Times of pregnancy (mean ± SE)	3.21 ± 0.02	3.00 ± 0.02	&lt; 0.01*	3.33 ± 0.06	3.08 ± 0.01	&lt; 0.01*
Abortion (%)	2329(67.8)	2652(67.8)	0.95	413(65.8)	4568(68.0)	0.25
Oral contraceptives use (%)	656(18.9)	672(16.9)	0.02*	129(20.4)	1199(17.6)	0.08
Years of post-menopause (mean ± SD)	13.8 ± 9.3	11.9 ± 9.2	&lt; 0.01*	15.8 ± 9.5	12.5 ± 9.2	&lt; 0.01*
History of HT (%)	126(3.9)	99(2.8)	0.01*	26(4.3)	199(3.2)	0.02*
Education (%)			&lt; 0.01*			0.36
Elementary or lower	807(23.2)	797(20.0)		154(24.1)	1450(21.2)	
Junior high school	1278(36.7)	1424(35.7)		229(35.8)	2473(36.2)	
High school	1071(30.8)	1379(34.5)		199(31.1)	2251(32.9)	
College or higher	325(9.3)	393(9.8)		57(8.9)	661(9.7)	
Alcohol consumption (%)			0.42			0.77
Never	3031(86.9)	3513(87.7)		557(87.2)	5987(87.4)	
Current	410(11.8)	433(10.8)		71(11.1)	772(11.2)	
Former	47(1.3)	58(1.5)		11(1.7)	94(1.4)	
Physical exercise (%)	3096(88.6)	3605(89.8)	0.07	577(90.1)	6124(89.2)	0.43
Work posture (%)			0.22			0.29
Sitting	1031(29.6)	1268(31.7)		207(32.5)	2092(30.6)	
Standing	1765(50.7)	1942(48.6)		320(50.2)	3387(49.5)	
Bending	535(15.4)	616(15.4)		90(14.1)	1061(15.5)	
Squatting or kneeling	150(4.3)	173(4.3)		20(3.1)	303(4.4)	
Shift work	1495(42.8)	1522(37.9)	&lt; 0.01*	264(41.2)	2753(40.1)	0.56

*BMI* body mass index, *HT* post-menopausal hormone replacement therapy, *SD* standard deviation, *SE* standard error, Continuous variables were described as the mean ± SD and compared by Student’s t-test, and categorical variables were described as number (percentage) and compared by Chi-square test. *p-value &lt; 0.05

As shown in Table 2, advanced age and elevated BMI were associated with an elevated prevalence of knee pain and clinical KOA. After adjusting for age and BMI, the ORs for knee pain and clinical KOA among women with ≥3 pregnancies were 1.38 (1.20–1.60) and 0.91 (0.69–1.21), respectively, when compared with those with ≤1 pregnancy. There were 1328 participants (17.7%) that reported taking oral contraceptives during their fertility period. Participants who had taken oral contraceptives had a higher prevalence of knee pain (OR 1.18, 95% CI 1.05–1.34) and clinical KOA (1.28, 1.04–1.57) than those who had never used oral contraceptives. Compared with women of pre-menopausal status, a significantly increased prevalence of knee pain was observed among post-menopausal women. The ORs for knee pain among women with &lt; 9, 9–17, and ≥ 17 years of post-menopausal status were 1.30 (1.08–1.56), 1.42 (1.16–1.76), and 1.46 (1.12–1.91), respectively. The corresponding prevalence for clinical KOA was increased, although not statistically significantly, with ORs of 1.33 (0.88–2.01), 1.35 (0.87–2.09), and 1.38 (0.82–2.32), respectively. HT was significantly associated with the increased prevalence of knee pain (1.59, 1.23–2.06) and clinical KOA (1.79, 1.21–2.65) among post-menopausal women. Further adjustments for education, physical exercise, alcohol drinking, work posture, and shift work did not change these associations.

**Table 2:** ORs and 95% CI of risk factors for knee pain and clinical KOA (logistic regression)

Variables	Knee pain				Clinical KOA			
	case	control	Model 1	Model 2	case	control	Model 1	Model 2
Age (years)	NA	NA	1.03(1.02–1.03)*		NA	NA	1.04(1.03–1.05)*	
BMI (kg/m^2^)	NA	NA	1.08(1.07–1.10)*		NA	NA	1.10(1.08–1.12)*	
Education								
Elementary or lower	807	797	1		154	1450	1	
Junior high school	1278	1424	1.06(0.93–1.21)		229	2473	1.20(0.96–1.51)	
High school	1071	1379	1.03(0.89–1.18)		199	2251	1.39(1.09–1.77)*	
College or higher	325	393	1.05(0.87–1.26)		57	661	1.21(0.87–1.67)	
Alcohol consumption								
Never	3031	3513	1		557	2987	1	
Current	410	433	1.21(1.04–1.40)*		71	772	1.18(0.91–1.54)	
Former	47	58	0.99(0.67–1.48)		11	94	1.42(0.75–2.70)	
Physical exercise								
No	400	408	1		63	745	1	
Yes	3096	3605	0.89(0.77–1.03)		577	6124	1.12(0.85–1.47)	
Work posture								
Sitting	1031	1268	1		207	2092	1	
Standing	1765	1942	1.12(1.01–1.24)*		320	3387	0.96(0.80–1.15)	
Bending	535	616	1.07(0.93–1.23)		90	1061	0.86(0.66–1.11)	
Squatting kneeling	150	173	1.07(0.84–1.35)		20	303	0.67(0.42–1.07)	
Shift work								
No	2001	2492	1		376	4117	1	
Yes	1495	1522	1.28(1.17–1.41)*		264	2753	1.13(0.96–1.34)	
Times of pregnancy								
≤1	387	553	1	1	67	873	1	1
2	774	1050	1.05(0.90–1.24)	0.97(0.82–1.15)	134	1690	0.90(0.66–1.23)	0.97(0.70–1.34)
≥3	2335	2411	1.38(1.20–1.60)*	1.15(0.98–1.34)	439	4307	0.91(0.69–1.21)	0.98(0.73–1.32)
Abortion								
No	1104	1261	1	1	215	2150	1	1
Yes	2329	2652	1.01(0.91–1.11)	1.08(0.98–1.20)	413	4568	1.06(0.88–1.26)	1.01(0.85–1.22)
History of HT								
No	3088	3418	1	1	580	5926	1	1
Yes	126	99	1.59(1.23–2.06)*	1.56(1.20–2.04)*	26	199	1.79(1.21–2.65)*	1.65(1.11–2.47)*
Years of post-menopause								
No	239	444	1	1	29	654	1	1
&lt;9	887	1208	1.30(1.08–1.56)*	1.31(1.08–1.58)*	134	1961	1.33(0.88–2.01)	1.36(0.89–2.09)
7–19	1065	1161	1.42(1.16–1.76)*	1.48(1.19–1.83)*	187	2039	1.35(0.87–2.09)	1.38(0.89–2.16)
≥17	1262	1148	1.46(1.12–1.91)*	1.54(1.17–2.01)*	285	2125	1.38(0.82–2.32)	1.39(0.82–2.35)
Contraceptives use								
No	2813	3307	1	1	504	5616	1	1
Yes	656	672	1.18(1.05–1.34)*	1.17(1.04–1.33)*	129	1199	1.28(1.04–1.57)*	1.25(1.01–1.54)*

*BMI* body mass index, *HT* post-menopausal hormone replacement therapy, *NA* not applicable, *p-value &lt; 0.05

Model 1: adjusted for age (continuous) and BMI (continuous)

Model 2: adjusted for age (continuous), BMI (continuous), education, alcohol drinking, physical exercise, work posture, and shift work

As shown in Table 3, the results of stepwise multivariable logistic regression analysis indicated that &lt; 9 years (1.32, 1.09–1.59), 9–17 years (1.46, 1.18–1.80), and ≥ 17 years (1.47, 1.12–1.93) post-menopause, oral contraceptives use (1.14; 1.01–1.29), HT (1.52; 1.16–1.98), shift work (1.28; 1.16–1.41), advanced age (1.01; 1.00–1.02), and elevated BMI (1.08; 1.06–1.09) were independently positively associated with an increased prevalence of knee pain. In addition, use of oral contraceptives (1.26; 1.02–1.55), HT (1.66; 1.12–2.48), aging (1.04; 1.03–1.05), and obesity (1.09; 1.07–1.12) were independently positively associated with an increased prevalence of clinical KOA.

**Table 3:** ORs of risk factors for knee pain and clinical KOA (multivariable stepwise logistic regression)

Variables	Knee pain	Clinical KOA
Times of pregnancy		
≤ 1	1	NA
2	0.95(0.80–1.13)	NA
≥ 3	1.12(0.96–1.31)	NA
Duration of post-menopause (years)		
No	1	NA
&lt; 9	1.32(1.09–1.59)*	NA
9–17	1.46(1.18–1.80)*	NA
≥ 17	1.47(1.12–1.93)*	NA
History of HT	1.52(1.16–1.98)*	1.66(1.12–2.48)*
Contraceptives use	1.14(1.01–1.29)*	1.26(1.02–1.55)*
Age (years)	1.01(1.00–1.02)*	1.04(1.03–1.05)*
BMI (kg/m^2^)	1.08(1.06–1.09)*	1.09(1.07–1.12)*
Shift work	1.28(1.16–1.41)*	NA

*BMI* body mass index, *HT* post-menopausal hormone replacement therapy, *NA* not applicable, and the variable was insignificantly associated with clinical KOA in the multivariable stepwise logistic regression analysis. The model initially included times of pregnancy, abortion, post-menopause duration, HT, contraceptives use, age, BMI, education, physical exercise, drinking, work posture, and shift work. *p-value &lt; 0.05

Table 4 showed the joint effects of obesity (BMI ≥24.0 kg/m^2^) and reproductive and hormone factors on knee pain and KOA. The results showed multiplicative interactions (*p* = 0.019) and additive (AP 0.26; 95% CI 0.11–0.42) effects of obesity and oral contraceptives use on knee pain. Compared with women with a BMI &lt; 24.0 kg/m^2^ and no contraceptives use, the ORs of knee pain among women with a BMI &lt; 24.0 kg/m^2^ and contraceptives use, with a BMI ≥24.0 kg/m^2^ and no contraceptives use, and with a BMI ≥24.0 kg/m^2^ and contraceptives use were 1.03(0.87–1.22), 1.44(1.30–1.59), and 2.00(1.68–2.38), respectively. There was an additive effect of obesity and pregnancy on knee pain (AP 0.08; 95% CI 0.07–0.22) but no statistically significant combined effects of obesity and reproductive and hormone factors on clinical KOA.

**Table 4:** Interaction effects of obesity and reproductive and hormone factors on knee pain and clinical KOA

Variables	Knee pain			Clinical KOA		
	OR (95% CI)	Multiplicative interaction, *p*-value	Additive interaction, AP	OR (95% CI)	Multiplicative interaction, *p*-value	Additive interaction, AP
BMI + pregnancy		0.570	0.08(0.07, 0.22)*		0.946	−0.02(−0.31, 0.27)
BMI (−) pregnancy (&lt; 3)	1			1		
BMI (−) pregnancy (≥3)	1.16(1.01–1.33)*			1.00(0.76–1.33)		
BMI (+) pregnancy (&lt; 3)	1.48(1.27–1.73)*			1.65(1.24–2.21)*		
BMI (+) pregnancy (≥3)	1.78(1.55–2.04)*			1.63(1.26–2.12)*		
BMI + abortion		0.544	0.07(−0.08, 0.23)		0.288	−0.14(− 0.42, 0.15)
BMI (−) abortion (−)	1			1		
BMI (−) abortion (+)	1.06(0.92–1.23)			1.20(0.90–1.60)		
BMI (+) abortion (−)	1.48(1.25–1.74)*			1.90(1.42–2.55)*		
BMI (+) abortion (+)	1.66(1.44–1.92)*			1.85(1.40–2.42)*		
BMI + menopause		0.329	0.18(−0.05, 0.41)		0.298	−0.15(− 0.67, 0.38)
BMI (−) menopause (−)	1			1		
BMI (−) menopause (+)	1.20(0.93–1.54)			2.08(0.96–4.51)		
BMI (+) menopause (−)	1.32(0.94–1.85)			2.73(1.09–6.88)*		
BMI (+) menopause (+)	1.84(1.43–2.38)*			3.32(1.53–7.20)*		
BMI + HT		0.828	0.09(−0.33, 0.51)		0.916	0.20(− 0.33, 0.74)
BMI (−) HT (−)	1			1		
BMI (−) HT (+)	1.62(1.14–2.32)*			1.72(0.93–3.17)		
BMI (+) HT (−)	1.52(1.39–1.67)*			1.62(1.37–1.93)*		
BMI (+) HT (+)	2.36(1.62–3.44)*			2.95(1.77–4.92)*		
BMI + contraceptives use		0.019*	0.26(0.11–0.42)*		0.133	−0.16(−0.54, 0.22)
BMI (−) contraceptives (−)	1			1		
BMI (−) contraceptives (+)	1.03(0.87–1.22)			1.52(1.12–2.06)*		
BMI (+) contraceptives (−)	1.44(1.30–1.59)*			1.74(1.44–2.11)*		
BMI (+) contraceptives (+)	2.00(1.68–2.38)*			1.96(1.46–2.62)*		

*AP* attributable proportion of interaction, *HT*, post-menopausal hormone replacement therapy, BMI (+) ≥24.0 kg/m^2^, BMI (−) &lt; 24.0 kg/m^2^, Adjusted for age (continuous), education level, alcohol consumption, physical exercise, work posture, and shift work. *p-value &lt; 0.05

## Discussion

In the present study, HT, use of oral contraceptives, aging and elevated BMI were associated with an elevated prevalence of knee pain and clinical KOA among female participants. A high number of pregnancies and post-menopausal status were independently associated with knee pain. Additionally, the results indicated multiplicative and additive effects of obesity and taking oral contraceptives and additive effects of obesity and pregnancy on knee pain among females.

Post-menopause status and estrogen deficiency have been reported to be associated with an increased risk of KOA [22], but the effects of post-menopausal HT on KOA were contradictory. A cross-sectional study reported a protective effect of HT on KOA among women with a mean age of 53 years [23]. Results from a randomized controlled trial and from the Framingham Study did not observe a significant association between HT and KOA [24, 25]. The case-control study from Sandmark and colleagues suggested that HT increased the risk of KOA [26]. In the present study, HT was independently associated with an elevated prevalence of knee pain and clinical KOA among post-menopause women, with ORs of 1.52 (1.16–1.98) for knee pain and 1.66 (1.12–2.48) for clinical KOA. Several factors might contribute to the varied results. First, the dose and duration regarding the use of hormone replacement varied among researches. Second, although estrogen receptors are found in the musculoskeletal system, the effect of post-menopausal hormone supplement on cartilage and bone was uncertain [27–30]. Third, ethnic differences might partially explain the varied results. Among females using HT, American Indians ran a higher risk of OA than Asians and Whites [31]. In addition, we have noticed that education level was higher among those using HT (66.1% in high school or higher education) than those not taking HT (39.7% in high school or higher education), which might suggest different lifestyles among women using or not using HT [32].

Our results suggested that a high number of pregnancies was associated with an elevated prevalence of KOA, which is consistent with previous study results [9, 33, 34]. Women that had more pregnancies tended to have a higher BMI due to postpartum weight retention [35, 36], and elevated BMI was associated with an imbalance of bone and cartilage metabolism [37, 38].

In the present study, oral contraceptives use and obesity showed combined effects on KOA. Oral contraceptives were reported to be associated with venous thromboembolism [39–41], and the latter lead to subchondral ischemia and ultimately bone dystrophy. In addition, both oral contraceptives use and obesity were reported to increase plasma total cholesterol and triglyceride levels [42–44], and hyperlipidemia might be a potential risk factor for bone and cartilage metabolism disorders [45, 46].

The strengths of our study include its large sample size, detailed information regarding demographics, reproductive and hormone factors, medical history, and occupational history for each participant, and examinations of knee joints. However, the present study had several limitations. First, we failed to collect data of weight retention for each pregnancy and therefore we could not distinguish the effects of weight retention from pregnancy on KOA from the effect of total body weight change. Second, we were unable to determinate the estrogen levels of each participant and the association between hormone-related factors and KOA is unknown. Third, we did not provide knee X-ray examinations for the participants although all participants could undergo knee X-ray examinations at any time, which the company would pay for, if they experienced knee discomfort. Fourth, we estimated workload based on the job held for the longest duration. Participants might have had different jobs during their years of employment, but the workload during the shorter job durations was not considered.

## Conclusions

This study indicated that reproductive and hormone factors, such as a high number of pregnancies, oral contraceptives use, and HT, were associated with an elevated prevalence of knee pain and clinical KOA. In addition, obesity could increase the effects of reproductive and hormone factors on knee pain or clinical KOA among middle-aged and older Chinese women.

## Abbreviations

AP: Attributable proportion of interaction
BMI: Body mass index
CI: Confidence interval
HT: Hormone replacement therapy
KOA: Knee osteoarthritis
NA: not applicable
OR: Odds ratio
SD: Standard deviation
SE: Standard error
